# Revising Reverse-Phase Chromatographic Behavior for Efficient Differentiation of Both Positional and Geometrical Isomers of Dicaffeoylquinic Acids

**DOI:** 10.1155/2018/8694579

**Published:** 2018-01-11

**Authors:** Keabetswe Masike, Ian Dubery, Paul Steenkamp, Elize Smit, Edwin Madala

**Affiliations:** ^1^Department of Biochemistry, University of Johannesburg, P.O. Box 524, Auckland Park 2006, South Africa; ^2^CSIR Biosciences, Natural Products and Agro-Processing Group, Pretoria 0001, South Africa; ^3^Department of Chemistry, University of Johannesburg, P.O. Box 524, Auckland Park 2006, South Africa

## Abstract

Dicaffeoylquinic acids (diCQAs) are plant metabolites and undergo *trans*-*cis*-isomerization when exposed to UV irradiation. As such, diCQAs exist in both *trans*- and *cis*-configurations and amplify the already complex plant metabolome. However, analytical differentiation of these geometrical isomers using mass spectrometry (MS) approaches has proven to be extremely challenging. Exploring the chromatographic space to develop possible conditions that would aid in differentially separating and determining the elution order of these isomers is therefore imperative. In this study, simple chromatographic parameters, such as column chemistry (phenyl versus alkyl), mobile phase composition (methanol or acetonitrile), and column temperature, were investigated to aid in the separation of diCQA geometrical isomers. The high-performance liquid chromatography photodiode array (HPLC-PDA) chromatograms revealed four isomers post UV irradiation of diCQA authentic standards. The elution profile/order was seen to vary on different reverse-phase column chemistries (phenyl versus alkyl) using different mobile phase composition. Here, the elution profile/order on the phenyl-derived column matrices (with methanol as the mobile phase composition) was observed to be relatively reproducible as compared to the alkyl (C_18_) columns. Chromatographic resolution of diCQA geometrical isomers can be enhanced with an increase in column temperature. Lastly, the study highlights that chromatographic elution order/profile cannot be relied upon to fathom the complexity of isomeric plant metabolites.

## 1. Introduction

Dicaffeoylquinic acids (diCQAs) are plant secondary metabolites that are part of the family of bioactive metabolites called chlorogenic acids. Dicaffeoylquinic acids (diCQAs) are formed from an esterification reaction between quinic acid and 2 units of the hydroxycinnamic acid (HCA) derivative, caffeic acid [1, 2]. It is reported that HCA derivatives such as caffeic acid are initially synthesized in the *trans*-configuration through the phenylpropanoid pathway [3]. However, due to the 1,2-disubstituted alkenic molecular structure (carbon-carbon double bond), these molecules absorb light (UV light) at a specific wavelength and readily convert to the *cis*-geometry [3–7].

Previously, *cis*-isomers of phenylpropanoid derivatives were understood to exist in minor quantities and, as such, were thought to be biologically insignificant [3]. However, due to environmental changes, these isomers may be prominent in plant organs (i.e., leaves and fruits) that are exposed to the sun's UV rays [6] and, in some cases, exist in equal proportions to the *trans*-isomer. These new photochemically produced products (*cis*-isomers) amplify the already complex plant metabolome. Thus, studies devoted to exploring the biological significance of *cis*-isomers relative to their *trans*-counterparts have become necessary [8–13]. For instance, it has been shown that the phenylpropanoid pathway metabolite, *trans*-cinnamic acid, has less potent antituberculosis activity than its *cis*-counterpart, which is approximately 120-fold more than the *trans*-form [8]. In addition, in vacuo studies have demonstrated that HCA derivatives, such as dicaffeoyltartaric acid (chicoric acid) and diCQA, possess anti-human immunodeficiency virus (HIV) type 1 DNA integrase activity, with the biological activity attributed to their *cis*-isomers [10–12]. However, currently, only a few in vitro and in vivo studies, that show the biological activities of *cis*-isomers of HCA derivatives, exist. This is possibly due to the lack of knowledge about the existence of *cis-*isomers of HCA derivatives or the lack of *cis*-form commercial standards [3].

**Scheme 1:**
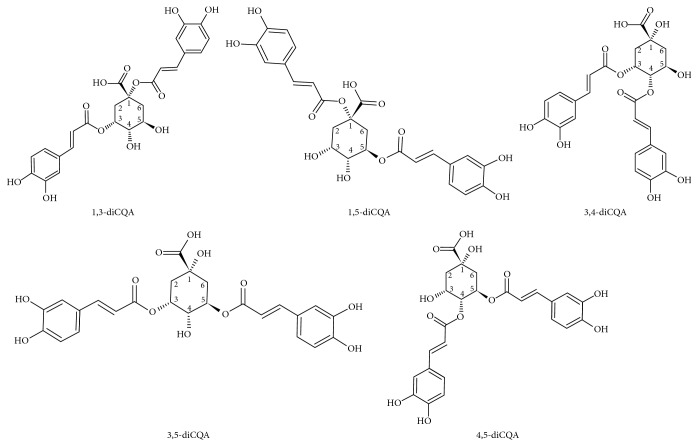
Chemical structures of the *trans*-form positional isomers of dicaffeoylquinic acids (diCQAs), namely, 1,3-diCQA, 1,5-diCQA, 3,4-diCQA, 3,5-diCQA, and 4,5-diCQA.

Authentic standards of most of the HCA derivatives (*trans*-isomers), such as diCQAs, are commercially available, and these standards can be used to produce their *cis*-counterparts through the process of photoisomerization [4, 6, 12, 14, 15]. Identification of these related geometrical compounds using analytical techniques such as liquid chromatography linked to mass spectrometry (LC-MS) has proven impossible as they produce similar/identical MS fragmentation patterns [4]. As such, these analytical challenges have driven efforts in exploring the chromatographic space to suggest possible conditions that would aid in differentially separating and identifying these isomers [4, 12]. In this endeavor, Clifford et al. UV irradiated five different authentic standards of diCQA positional isomers, namely, 1,3-diCQA, 1,5-diCQA, 3,4-diCQA, 3,5-diCQA, and 4,5-diCQA, and analyzed the samples on a phenyl-hexyl column using aqueous acetonitrile as part of the mobile phase composition [4]. From the study, Clifford et al. anticipated three possible *cis*-isomers for all five of the diCQA positional isomers. The number of *cis*-isomers can be attributed to the stereochemistry of the quinic acid unit (positions 1, 3, 4, and 5) to which the caffeic acid units are esterified [4, 14] (Scheme 1). As such, two asymmetrical mono-*cis*-isomers (resulting from *cis*-isomerization on the respective caffeoyl arms on the quinic acid unit) and one di-*cis*-isomer (resulting from the *cis*-geometry on both caffeoyl arms on the quinic acid unit) were anticipated for each diCQA positional isomer. However, in the study by Clifford et al., only two (instead of three) *cis*-isomers were observed for 1,3-diCQA, 3,4-diCQA, and 3,5-diCQA positional isomers [4].

Fundamentally, column chemistry [12, 16, 17], mobile phase composition [12, 16], and column temperature [18, 19] are the essential factors in defining chromatographic separation during LC analyses. For instance, under reverse-phase conditions, a phenyl-derived column matrix may produce a different elution profile (i.e., number of isomers separated) compared to an alkyl-derived column matrix, due to the interactions involved in the retention mechanism of the respective columns [12, 16].

The aims of the current study were to reproduce and expand on the results observed by Clifford et al. [4]. In our study, the abovementioned positional isomers of diCQAs (1,3-diCQA, 1,5-diCQA, 3,4-diCQA, 3,5-diCQA, and 4,5-diCQA) were UV irradiated, and the resulting samples were analyzed on nine different column chemistries, five phenyl-derived columns and four alkyl (C_18_) columns. The effect of chromatographic parameters (column choice, mobile phase composition, and column temperature) on the chromatographic separation of the UV-generated diCQA geometrical isomers was evaluated. Insight on the chromatographic elution order of these metabolites (both *trans*- and *cis*-isomers) will contribute to ongoing efforts in designing analytical methods for differential identification of isomers contributing to plant sample dimensionality. Such chromatographic separation efforts will further allow collection (LC fractionation) of these peaks (metabolites) to study their respective bioactivity differences.

## 2. Materials and Methods

### 2.1. Materials

Authentic standards (with the purity of above 99.6%) of *trans*-dicaffeoylquinic acid (1,3-diCQA, 1,5-diCQA, 3,4-diCQA, 3,5-diCQA, and 4,5-diCQA) were purchased from Phytolab (Vestenbergsgreuth, Germany). Mass spectrometry grade (99.9%) methanol and acetonitrile were obtained from Romil (Cambridge, UK). Mass spectrometry grade formic acid was purchased from Sigma-Aldrich (St. Louis, MO, USA). A UV light box Model CM-10 was purchased from Spectroline (Westbury, NY, USA). Pinnacle, Raptor, Viva, and Ultra columns were purchased from Restek (Bellefonte, PA, USA) and Kinetex columns were purchased from Phenomenex (Torrance, CA, USA). Chromatographic separation was achieved using nine analytical columns: Pinnacle bi-phenyl and C_18_ (2.1 × 100 mm, 3 µm), Raptor bi-phenyl and C_18_ (2.1 × 100 mm, 2.7 µm), Viva bi-phenyl (2.1 × 100 mm, 5 µm), Viva C_18_ (2.1 × 100 mm, 3 µm), Ultra C_18_ (2.1 × 100 mm, 3 µm), and Kinetex bi-phenyl and phenyl-hexyl (4.6 × 100 mm, 5 µm).

### 2.2. Methods

#### 2.2.1. UV Irradiation

A 1 mg/mL solution of each *trans*-diCQA positional isomer was prepared with 100% methanol. Irradiation of samples was conducted following the procedure described elsewhere [15]. Briefly, the solution (for each positional isomer) was placed in a Spectroline UV lamp operating at 254 nm with an intensity of 390 μW/cm^2^. The lamp was not covered with any notch filter. Irradiation was conducted for four hours (4 h), and aliquots (100 µL) were taken at 0 h (before irradiation) and at 4 h post irradiation. The aliquots were diluted 10 × with 100% methanol. All the samples were placed in amber vials and subjected to HPLC-PDA analyses.

#### 2.2.2. HPLC-PDA Parameters

The HPLC system used was a Shimadzu SCL-10A VP (Kyoto, Japan), equipped with a PDA controlled by Shimadzu VP software v. 5.31. Column oven temperature was set at 30°C and 50°C. The injection volume was 3 µL. A binary solvent mixture was used, consisting of Milli-Q water (eluent A) containing 0.1% formic acid and methanol or acetonitrile (eluent B) containing 0.1% formic acid. The initial conditions were 10% B at a flow rate of 0.2 mL/min and were maintained for 1 min, followed by an increase to 40% B at 15 min; the conditions were maintained for 2 min, followed by multiple gradients to 90% at 20 min; and the conditions were kept constant for 3 min and then changed to the initial conditions (10% B) after 5 min, followed by a 7-min isocratic wash at 10% B to re-equilibrate the column. The total chromatographic run time was 35 min. The PDA detector scanning range was set from 220 to 400 nm, and the chromatograms were processed at 325 nm. A column temperature study was conducted using a longer LC program (45 mins) to enhance the separation of diCQA geometrical isomers. The column oven temperature was set at 30, 35, 40, 45, 50, 55, and 60°C.

## 3. Results and Discussion

In this study, positional isomers of diCQAs (Scheme 1) were UV irradiated, and the resulting samples were analyzed under reverse-phase chromatographic conditions using nine different column chemistries. Here, for each column, either methanol (containing 0.1% formic acid) or acetonitrile (containing 0.1% formic acid) was used as the mobile phase/eluent B, with the initial column oven temperature set at 30 and then 50°C. Following analyses of the results, the use of aqueous methanol as the mobile phase/eluent B showed enhanced separation and, as such, the results discussed herein will be those obtained with methanol as part of the mobile phase composition [12]. The enhanced chromatographic separation using methanol as part of the binary solvent mixture is due to the weak eluent nature of methanol, which enhances the chromatographic separation of aromatic compounds by promoting longer retention within the column [12, 20]. However, the results obtained with aqueous acetonitrile will also be referred to when necessary. In addition, unless stated otherwise, the results discussed herein are those obtained with the column oven temperature set at 30°C.

**Figure 1:**
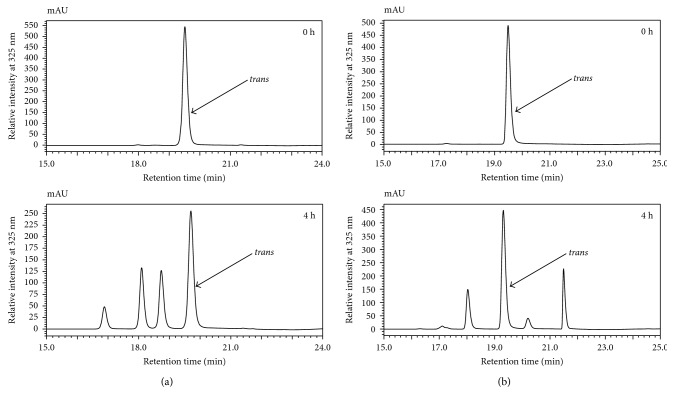
Comparisons of HPLC-PDA chromatogram profiles of nonirradiated (0 h) and UV irradiated (4 h) samples of (a) 3,5-diCQA analyzed on a Pinnacle C_18_ column and (b) 4,5-diCQA analyzed on a Viva C_18_ column, using aqueous methanol as the mobile phase composition.

The chromatograms of the nonirradiated (0 h) and irradiated (4 h) samples were compared and the retention times (*t*_*R*_) of the peak observed from the nonirradiated samples were used to identify the *trans*-isomers from each irradiated diCQA sample, for each column used to conduct the study (Figure 1). A summary of the chromatographic results is represented in Table 1, where the void volume/“dead time” was assessed for each column by the inspection of the chromatograms, and the resulting capacity factors (*k*) for the *trans*- and *cis*-isomers are shown.

### 3.1. The Effect of Column Chemistry on the Separation of diCQA Geometrical Isomers

**Figure 2:**
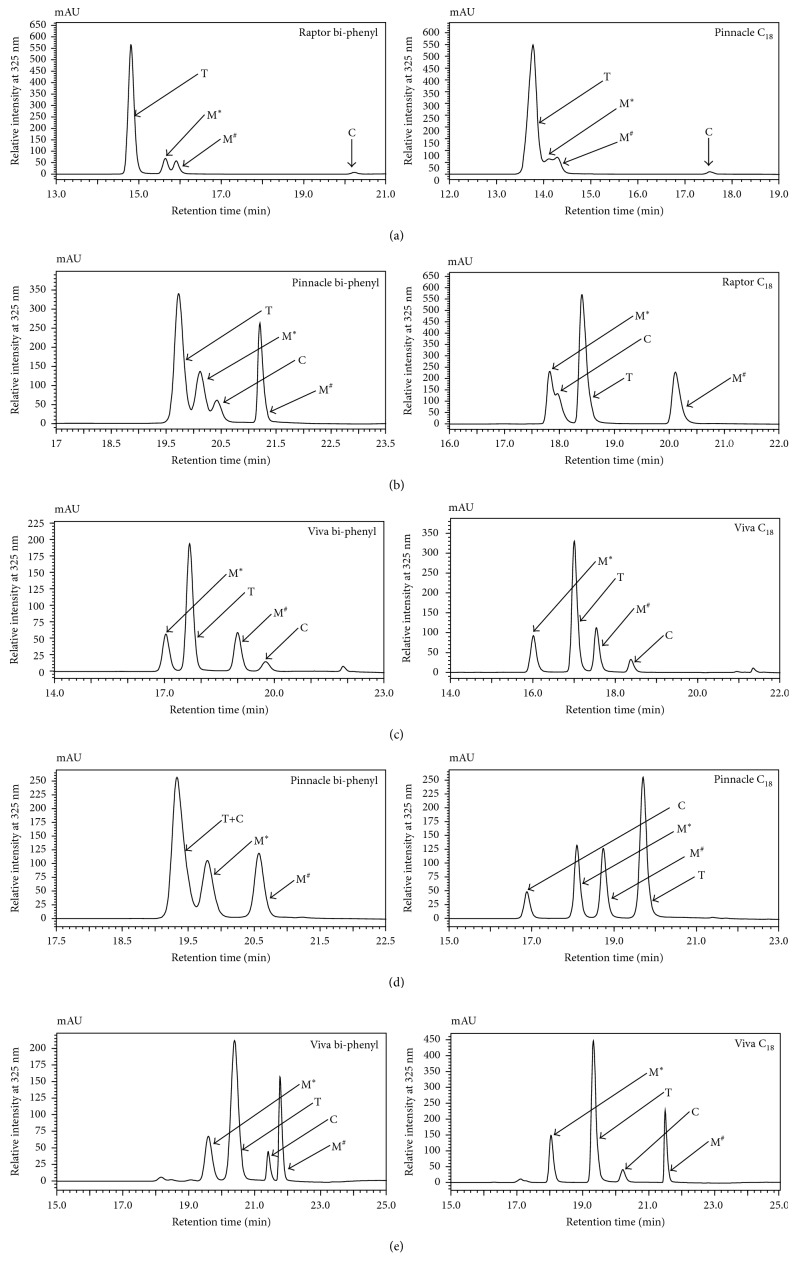
Overlaid HPLC-PDA chromatograms demonstrating the chromatographic separation of UV-irradiated samples of (a) 1,3-diCQA, (b) 1,5-diCQA, (c) 3,4-diCQA, (d) 3,5-diCQA, and (e) 4,5-diCQA analyzed on either a bi-phenyl or C_18_ column matrix. The figure shows differences and similarities in the elution profiles, dependent on either the bi-phenyl or C_18_ column matrix. **T** represents the di-*trans*-isomer, **M** ^∗^ represents the first eluting mono-*cis*-isomer, **M** ^**#**^ represents the second eluting mono-*cis*-isomer, and **C** represents the di-*cis*-isomer.

The results show that the chromatographic profile for the UV-irradiated sample of 1,3-diCQA is consistent on both the bi-phenyl and C_18_ column matrices (Figure 2(a)) and consistent with the results achieved by Clifford et al. [4]. On both the bi-phenyl and C_18_ column matrices (Figure 2(a)), two mono-*cis*-isomers (**M** ^∗^ and **M** ^**#**^) were observed to elute after their respective *trans*-counterpart (**T**). According to Clifford et al., a peak of minor intensity is considered the di-*cis*-isomer [4]; thus, from Figure 2(a), **C** was annotated as the di-*cis*-isomer. In a study by Zheng et al., where the 3,5-diCQA geometrical isomers were separated by ion mobility, the photoisomerization study revealed that the di-*cis*-isomer forms directly from both the mono-*cis*-isomers [15]. In this study, the di-*cis*-isomer was retained longer on both column matrices (bi-phenyl and C_18_), suggesting the resolution of the di-*cis*-isomer from the other isomers (Figure 2(a)). Thus, for simplicity, the elution order of the various isomers for 1,3-diCQA is referred to as **TM** ^****^^∗^ **M** ^**#**^ **C**, where for all diCQAs, **T** represents the di-*trans*, **M** ^∗^ represents the first eluting mono-*cis*-isomer, **M** ^**#**^ represents the second eluting mono-*cis*-isomer, and **C** represents the di-*cis*-isomer. Furthermore, the elution profile/order for all diCQAs is summarized in Table 2.

**Figure 3:**
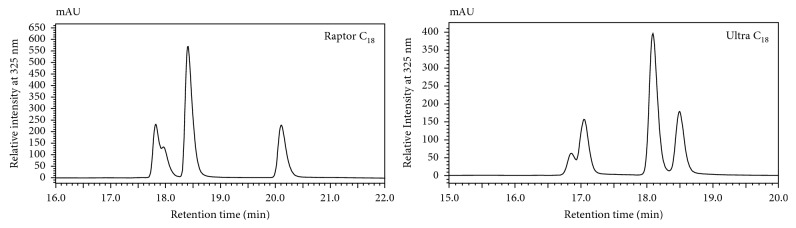
HPLC-PDA chromatograms of the UV-irradiated sample of 1,5-diCQA, showing different elution profiles on C_18_ column matrices. The Raptor C_18_ shows the elution profile **M** ^∗^ **CTM** ^**#**^, and the Ultra C_18_ shows the elution profile **CM** ^∗^ **TM** ^**#**^.

For the UV-irradiated sample of 1,5-diCQA, four peaks (corresponding to the four isomers) were observed when using the bi-phenyl and C_18_ column matrices, and it is apparent that the elution profile/order differs between the two column matrices (Figure 2(b)). On the C_18_ column matrix, two peaks, the first mono-*cis*-isomer (**M** ^∗^) and the di-*cis*-isomer (**C**), elute before the *trans*-isomer (**T**) and the fourth peak, the second mono-*cis*-isomer (**M** ^**#**^), elutes after the di-*trans*-isomer, resulting in the elution order **M** ^∗^ **CTM** ^**#**^ (Table 2). However, on the bi-phenyl column, the *cis*-isomers are seen to elute after the *trans*-isomer, resulting with the elution order **TM** ^∗^ **CM** ^**#**^ (Figure 2(b)) (Table 2). The elution order, **TM** ^∗^ **CM** ^**#**^, observed on the bi-phenyl columns was similar to the elution order observed by Clifford et al., using a phenyl-hexyl column matrix [4]. The consistency observed on the phenyl-containing column matrices (bi-phenyl versus phenyl-hexyl) suggests the possible role of *π-π* interactions in the separation of these aromatic isomers [12, 16, 17, 21]. In contrast, differences in the elution order amongst the C_18_ columns were observed (Figure 3). Instead of the elution order **M** ^∗^ **CTM** ^**#**^ seen in Figure 2(b) using a Raptor C_18_, the Ultra C_18_ column produced the elution order **CM** ^∗^ **TM** ^**#**^ (Figure 3).

The elution order observed for the UV-irradiated standard of 3,4-diCQA demonstrated a relatively identical elution profile, on columns showing enhanced separation of the four isomers. Where the first mono-*cis*-isomer (**M** ^∗^) elutes before the *trans*-isomer (**T**), the *trans*-isomer is followed by the second mono-*cis*-isomer (**M** ^**#**^) and lastly followed by the di-*cis*-isomer (**C**), resulting in the elution order **M** ^∗^ **TM** ^**#**^ **C** (Figure 2(c)) (Table 2). The similar elution order observed on both the bi-phenyl and C_18_ column matrices can be attributed to the stereochemistry at positions 3 and 4 on the quinic acid unit (Scheme 1, Figure 2(c)). The similarities in the spatial arrangement of the caffeic acid units at these positions on the quinic acid unit could possibly result in comparable interactions with their surrounding environments (i.e., mobile and stationary phase). In addition, the similar elution profile seen on both the bi-phenyl and C_18_ column matrices for the UV-irradiated sample of 1,3-diCQA (**TM** ^∗^ **M** ^**#**^ **C**) (Figure 2(a), Table 2) can also be attributed to the identical spatial arrangement at positions 1 and 3 on the quinic acid unit to which the caffeic acid units are esterified. Furthermore, in their study, Clifford et al. only observed three geometrical isomers (instead of four) for the UV-irradiated sample of 3,4-diCQA using a phenyl-hexyl column [4]. Here, the elution order observed was **M** ^∗^ **TM** ^**#**^ instead of the elution order **M** ^∗^ **TM** ^**#**^ **C** observed in our study and discussed above. Results observed by Clifford et al. were also observed in our study when the phenyl-hexyl column was used with aqueous acetonitrile as the eluent (see Supplementary Figure S1), thus suggesting the possible coelution of the di-*cis*-isomer under these conditions.

When the UV-generated geometrical isomers of 3,5-diCQA were analyzed on the phenyl-containing column matrices (at a column temperature of 30°C), only three peaks (instead of four peaks) were observed and resulted with the elution order **TM** ^∗^ **M** ^**#**^ (Figure 2(d), Table 2), as observed by Clifford et al. [4]. On the C_18_ column matrices, the elution order **CM** ^∗^ **M** ^**#**^ **T** was observed (Figure 2(d), Table 2).

**Figure 4:**
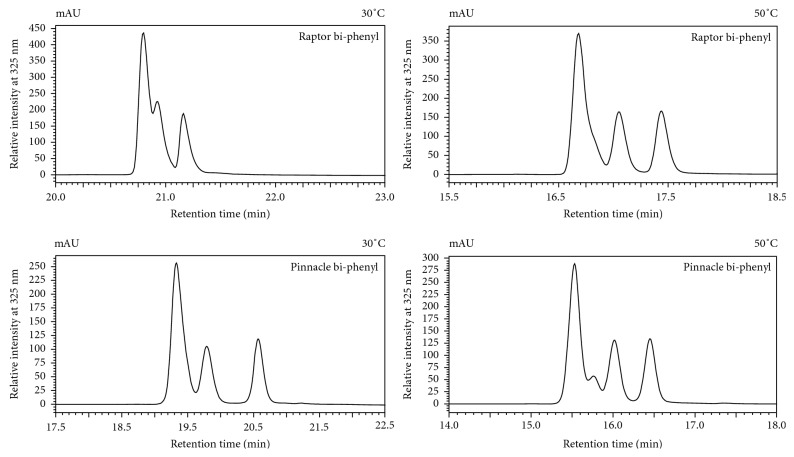
Comparison of HPLC-PDA chromatograms of UV-irradiated sample of 3,5-diCQA achieved with bi-phenyl columns, showing the enhanced separation of isomers due to an increase in column temperature.

Although not commonly considered a key parameter in reverse-phase chromatography, high column temperatures have been shown to enhance the separation due to a decrease in the viscosity of the mobile phase. In addition, retention factors are dependent on the distribution coefficients (*k*_*d*_) of the analytes, which are temperature dependent. An increase in temperature enhances the separation [19, 22, 23]. Thus, to achieve the separation of 3,5-diCQA geometrical isomers on the phenyl-containing column matrices, a column temperature of 50°C was introduced and resulted in the elution order **TCM** ^∗^ **M** ^**#**^ (Figure 4) instead of the elution order **TM** ^∗^ **M** ^**#**^ seen in Figure 2(d). The consistency in the elution profile observed on the phenyl column matrices (**TM** ^∗^ **M** ^**#**^) can be attributed to *π-π* interactions which enhance separation in phenyl-containing columns [12, 16, 17, 20, 21], and the elution profile differences observed between column matrices (phenyl versus alkyl matrices) can be attributed to the differences in spatial arrangements of the caffeoyl units at positions 3 and 5 on the quinic acid unit. For instance, the different spatial arrangements at these positions affect when *cis*-isomers elute on a phenyl column matrix (elutes after the *trans*-isomer; **TCM** ^∗^ **M** ^**#**^) versus an alkyl column (elutes before the *trans*-isomer; **CM** ^∗^ **M** ^**#**^ **T**).

**Figure 5:**
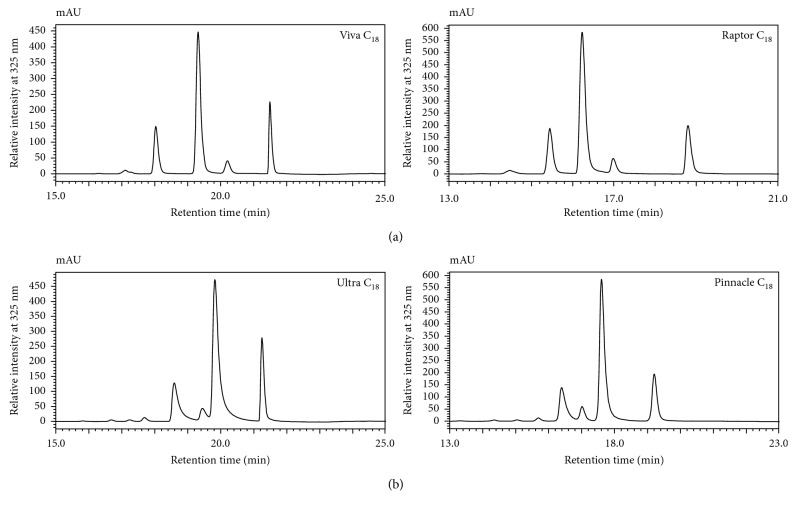
HPLC-PDA chromatograms of UV-irradiated sample of 4,5-diCQA showing different elution profiles, (a) **M** ^∗^ **TCM** ^**#**^ and (b) **M** ^∗^ **CTM** ^**#**^, on four C_18_ column matrices.

Finally, the elution order for the geometrical isomers of 4,5-diCQA was seen to be **M** ^∗^ **TCM** ^**#**^, only on three bi-phenyl columns and two C_18_ columns as summarized in Table 2 and demonstrated in Figure 2(e). Interestingly, the other two C_18_ columns showed a different elution order (Figure 5); thus, instead of **M** ^∗^ **TCM** ^**#**^ (Figure 5(a)), **M** ^∗^ **CTM** ^**#**^ was observed (Figure 5(b)). Furthermore, for this sample, Clifford et al. observed the elution order **TM** ^∗^ **CM** ^**#**^ when using a phenyl-hexyl column [4]. In our study, these results were also observed using a Phenomenex bi-phenyl column with acetonitrile as part of the mobile phase composition (Supplementary data, Figure S2). Thus, care must be taken when analyzing geometrical isomers of 4,5-diCQA on different column matrices.

Despite the inconsistent chromatographic elution profiles observed for the UV-irradiated sample of 4,5-diCQA, what seems to be consistent is the later elution of the second eluting mono-*cis*-isomer (**M** ^**#**^) (Figure 5). This is also evident in samples of 1,5-diCQA (Figure 2(b)) and 3,5-diCQA (Figure 2(d), Figure 4), especially when analyzed on phenyl-containing column matrices. This mono-*cis*-isomer could possibly be a *cis*-isomer at position 5 on the quinic acid unit. According to Clifford et al., a *cis*-geometry at position 5 on the quinic acid for mono-acyl chlorogenic acids results in intramolecular hydrogen bonding at two positions; (1) the caffeoyl carbonyl (C=O) group and the 4-OH group on the quinic acid unit and (2) the caffeoyl 3′-OH group and the carbonyl group at position 1 on the quinic acid unit [4], thus rendering the molecules less hydrophilic due to unavailable hydroxyl groups and the compact nature of the molecule. In this study, it is uncertain to what extent the above applies to the diCQAs (Supplementary data, Figure S3).

### 3.2. The Effect of Column Temperature on the Separation of diCQA Geometrical Isomers

**Figure 6:**
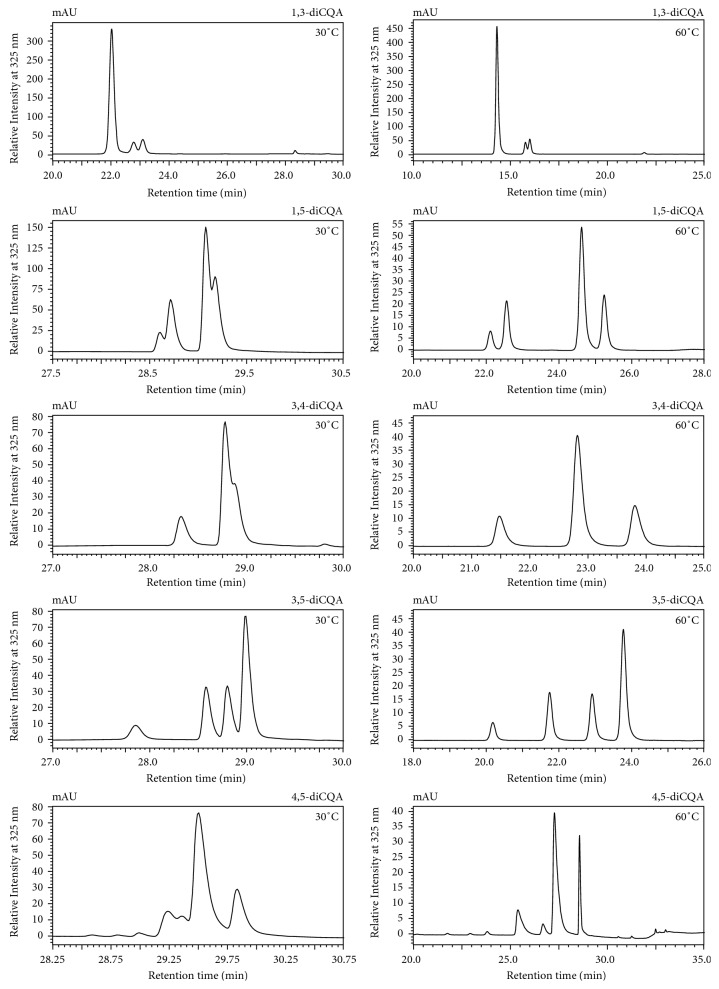
Graphical representation of the influence of temperature in enhancing the resolution of diCQA geometrical isomers as indicated. The figure shows that an increase in column temperature enhances the separation of diCQA geometrical isomers.

From the above results, it is apparent that determining the elution order of diCQA geometrical isomers on different reverse-phase column matrices shows inconsistencies (Table 2). Within the C_18_ column matrices, different chromatographic elution profiles were observed, and the Ultra C_18_ column showed the worst performance when analysis was conducted with the column temperature set at 30°C. As such, column temperature was varied (30–60°C) to enhance the separation of the diCQA geometrical isomers on the Ultra C_18_ column (Figure 6). From Figure 6, an increase in column temperature showed a positive effect on the resolution of the geometrical isomers of 1,3-diCQA, 1,5-diCQA, 3,5-diCQA, and 4,5-diCQA. An increase in temperature resulted in the earlier elution of analytes and resolution of the UV-irradiated diCQA geometrical isomers (Figure 6). Similar results were observed by Nguyen et al., whereby a pharmaceutical cocktail was chromatographically separated at temperatures 30°C and 90°C, and the temperature at 90°C enhanced the separation and decreased the analysis time [19]. Furthermore, in our study the UV-irradiated sample of 3,4-diCQA showed the separation and resolution of only three isomers, suggesting coelution of the di-*cis*-isomer. For this sample (UV-irradiated sample of 3,4-diCQA), the elution order (**M** ^∗^ **TM** ^**#**^) was also observed using the phenyl-hexyl column coupled with aqueous acetonitrile as part of the mobile phase (Supplementary data, Figure S4). The results obtained in this temperature study suggest that some column matrices are incapable of separating/distinguishing all the available isomers in the sample even post optimization of the chromatographic parameters.

## 4. Conclusion

This study demonstrates that positional isomers of diCQA samples produce three *cis*-isomers post UV irradiation, and separation of these isomers is dependent on optimization of primary chromatographic parameters. As such, column chemistry, mobile phase composition, and column temperature influence the chromatographic elution profile of the structurally related compounds, thus hindering identification. From the above results, it is apparent that determining the elution profile/order of diCQA geometrical isomers on different reverse-phase column matrices (phenyl versus alkyl) shows inconsistencies. However, a relatively consistent elution order was observed using the phenyl-containing column matrices, suggesting the important role of *π-π* interactions in the separation of the respective diCQA geometrical isomers. The results show different elution profiles between C_18_ column matrices from different column suppliers, suggesting that column manufacturing is not standardized.

The study also shows that column temperature can be used to enhance the separation of the isomers. Furthermore, the number of observed isomers depends on the capability of the column to distinguish the isomers. For instance, using the Ultra C_18_ column, the separation of the 3,4-diCQA geometrical isomers was enhanced by the introduction of column temperature; however, the di-*cis*-isomer was not observed on the chromatogram, suggesting coelution of the di-*cis*-isomer. Lastly, the study highlights that chromatographic elution order/profile cannot be relied upon to fathom the complexity of isomeric plant metabolites and that more advanced analytical methods need to be developed to achieve this goal. Advancement in analytical approaches can include hyphenation of high-temperature liquid chromatography (HTLC) to metal binding-based MS differentiation [14] or hyphenation of HTLC to ion mobility MS [15].

## Figures and Tables

**Table 1:** Capacity factors (*k*) of *trans*- and *cis*-isomers of diCQAs on different phenyl-derived and C_18_ column matrices.

Identity		Capacity factors (*k*′) (min)								
		Pinnacle bi-phenyl	Raptor bi-phenyl	Viva bi-phenyl	Phenomenex bi-phenyl	Phenomenex phenyl-hexyl	Pinnacle C_18_	Raptor C_18_	Viva C_18_	Ultra C_18_
1,3-diCQA	Di-*trans*	13.324–1.442	14.805–1.436	12.207–1.559	22.629–0.559	22.206–1.078	13.764–1.480	12.420–1.235	11.380–1.487	16.936–1.295
	Mono-*cis*	14.365–1.442	15.638–1.436	13.276–1.559	23.712–0.559	22.818–1.078	14.104–1.480	13.089–1.235	12.196–1.487	17.292–1.295
	Mono-*cis*	14.565–1.442	15.901–1.436	13.472–1.559	23.848–0.559	23.029–1.078	14.277–1.480	13.257–1.235	12.535–1.487	—
	Di-*cis*	19.168–1.442	20.217–1.436	18.112–1.559	25.560–0.559	26.050–1.078	17.523–1.480	17.546–1.235	17.231–1.487	20.918–1.295
1,5-diCQA	Di-*trans*	19.721–1.442	20.952–1.436	18.541–1.559	25.569–0.559	26.262–1.078	20.299–1.480	18.400–1.235	17.531–1.487	21.619–1.295
	Mono-*cis*	20.111–1.442	21.052–1.436	—	25.723–0.559	—	18.498–1.480	17.814–1.235	17.154–1.487	21.351–1.295
	Mono-*cis*	21.198–1.442	21.402–1.436	20.386–1.559	25.855–0.559	—	—	20.101–1.235	19.396–1.487	—
	Di-*cis*	20.417–1.442	—	—	—	—	18.035–1.480	17.955–1.235	—	—
3,4-diCQA	Di-*trans*	18.711–1.442	20.178–1.436	17.679–1.559	25.408–0.559	26.697–1.078	18.580–1.480	17.471–1.235	16.994–1.487	21.156–1.295
	Mono-*cis*	18.102–1.442	19.462–1.436	17.031–1.559	25.691–0.559	—	17.424–1.480	16.523–1.235	16.003–1.487	20.399–1.295
	Mono-*cis*	20.401–1.442	21.065–1.436	18.987–1.559	—	—	18.901–1.480	18.038–1.235	17.533–1.487	—
	Di-*cis*	20.984–1.442	21.248–1.436	19.753–1.559	—	—	—	18.706–1.235	18.370–1.487	—
3,5-diCQA	Di-*trans*	19.322–1.442	20.791–1.436	18.158–1.559	25.539–0.559	25.855–1.078	19.693–1.480	17.963–1.235	17.099–1.487	21.334–1.295
	Mono-*cis*	19.784–1.442	20.919–1.436	18.471–1.559	25.641–0.559	—	18.089–1.480	17.773–1.235	—	20.846–1.295
	Mono-*cis*	20.565–1.442	21.156–1.436	19.074–1.559	25.721–0.559	—	18.729–1.480	—	—	21.124–1.295
	Di-*cis*	—	—	—	—	—	16.877–1.480	16.945–1.235	16.304–1.487	19.561–1.295
4,5-diCQA	Di-*trans*	21.209–1.442	21.463–1.436	20.379–1.559	25.877–0.559	26.209–1.078	21.341–1.480	20.213–1.235	19.309–1.487	21.766–1.295
	Mono-*cis*	20.952–1.442	21.338–1.436	19.586–1.559	—	—	20.385–1.480	18.808–1.235	18.025–1.487	21.520–1.295
	Mono-*cis*	21.906–1.442	21.935–1.436	21.759–1.559	26.330–0.559	26.491–1.078	21.704–1.480	21.369–1.235	21.487–1.487	22.025–1.295
	Di-*cis*	21.654–1.442	21.702–1.436	21.399–1.559	26.163–0.559	—	20.947–1.480	20.638–1.235	20.200–1.487	—

**Table 2:** Elution order of the *trans*- and *cis*-isomers of diCQAs on different column matrices (phenyl versus alkyl) using aqueous methanol as the eluent post optimization.

		Column chemistry								
		Pinnacle bi-phenyl	Raptor bi-phenyl	Viva bi-phenyl	Phenomenex bi-phenyl	Phenomenex phenyl-hexyl	Pinnacle C_18_	Raptor C_18_	Viva C_18_	Ultra C_18_
Elution order of isomers	1,3-diCQA	**TM** ^∗^ **M** ^**#**^ **C**	**TM** ^∗^ **M** ^**#**^ **C**	**TM** ^∗^ **M** ^**#**^ **C**	**TM** ^∗^ **M** ^**#**^ **C**	**TM** ^∗^ **M** ^**#**^ **C**	**TM** ^∗^ **M** ^**#**^ **C**	**TM** ^∗^ **M** ^**#**^ **C**	**TM** ^∗^ **M** ^**#**^ **C**	**TM** ^∗^ **M** ^**#**^ **C**
	1,5-diCQA	**TM** ^∗^ **CM** ^#^	**TM** ^∗^ **CM** ^#^	**TM** ^∗^ **CM** ^#^	**TM** ^∗^ **M** ^#^	**TM** ^∗^	**CM** ^∗^ **T**	**M** ^∗^ **CTM** ^#^	**M** ^∗^ **TM** ^#^	**CM** ^∗^ **TM** ^#^
	3,4-diCQA	**M** ^∗^ **TM** ^#^ **C**	**M** ^∗^ **TM** ^#^ **C**	**M** ^∗^ **TM** ^#^ **C**	**TM** ^∗^ **C**	**M** ^∗^ **TM** ^#^	**M** ^∗^ **TCM** ^#^	**M** ^∗^ **TM** ^#^ **C**	**M** ^∗^ **TM** ^#^ **C**	**M** ^∗^ **TM** ^#^
	3,5-diCQA	**TCM** ^∗^ **M** ^#^	**TCM** ^∗^ **M** ^#^	**TCM** ^∗^ **M** ^#^	**TM** ^∗^ **M** ^#^	**T**	**CM** ^∗^ **M** ^#^ **T**	**CM** ^∗^ **T**	**CTM** ^#^	**CM** ^∗^ **M** ^#^ **T**
	4,5-diCQA	**M** ^∗^ **TCM** ^#^	**M** ^∗^ **TCM** ^#^	**M** ^∗^ **TCM** ^#^	**TM** ^∗^ **CM** ^#^	**M** ^∗^ **TM** ^#^	**M** ^∗^ **CTM** ^#^	**M** ^∗^ **TCM** ^#^	**M** ^∗^ **TCM** ^#^	**M** ^∗^ **CTM** ^#^

**T** represents the di-*trans*-isomer; **M** ^∗^ represents the first eluting mono-*cis*-isomer; **M** ^**#**^ represents the second eluting mono-*cis*-isomer; **C** represents the di-*cis*-isomer.

## Supplementary Materials

Supplementary Materials Figure S1: Elution profile of 3,4-diCQA geometrical isomers obtained using Phenomenex phenyl-hexyl and acetonitrile as the mobile phase composition. Figure S2: Elution profile of 4,5-diCQA geometrical isomers obtained using Phenomenex bi-phenyl and acetonitrile as the mobile phase composition. Figure S3: Energy optimized structure of geometrical isomers of 3,5 diCQA. The structures were optimized using the B3LYP/6-311+G(d,p) [11]. A) 3^trans^,5^trans^-diCQA (di-trans), B) 3^cis^,5^trans^-diCQA (mono-cis), C) 3^trans^,5^cis^-diCQA (mono-cis), and D) 3^cis^,5^cis^-diCQA (di-cis).
