# Supplementary material for: Revising Reverse-Phase Chromatographic Behavior for Efficient Differentiation of Both Positional and Geometrical Isomers of Dicaffeoylquinic Acids
Source: J Anal Methods Chem. 2018 Jan 11;2018:8694579. doi: 10.1155/2018/8694579 (PMC5821971; doi:10.1155/2018/8694579)
Supplement: Supplementary Materials — Figure S1: Elution profile of 3,4-diCQA geometrical isomers obtained using Phenomenex phenyl-hexyl and acetonitrile as the mobile phase composition. Figure S2: Elution profile of 4,5-diCQA geometrical isomers obtained using Phenomenex bi-phenyl and acetonitrile as the mobile phase composition. Figure S3: Energy optimized structure of geometrical isomers of 3,5 diCQA. The structures were optimized using the B3LYP/6-311+G(d,p) [11]. A) 3trans,5trans-diCQA (di-trans), B) 3cis,5trans-diCQA (mono-cis), C) 3trans,5cis-diCQA (mono-cis), and D) 3cis,5cis-diCQA (di-cis). [file 8694579.f1.docx]

**FIGURE LEGEND**

**Fig. S1.** Elution profile of 3,4-diCQA geometrical isomers obtained using Phenomenex phenyl-hexyl and acetonitrile as the mobile phase composition.

**Fig. S2.** Elution profile of 4,5-diCQA geometrical isomers obtained using Phenomenex bi-phenyl and acetonitrile as the mobile phase composition.

**Fig. S3.** Energy optimized structure of geometrical isomers of 3,5 diCQA. The structures were optimized using the B3LYP/6-311+G(d,p) [11]. **A**) 3^𝑡𝑟𝑎𝑛𝑠^,5^𝑡𝑟𝑎𝑛𝑠^-diCQA (di-trans), **B**) 3^𝑐𝑖𝑠^,5^𝑡𝑟𝑎𝑛𝑠^-diCQA (mono-cis), **C**) 3^𝑡𝑟𝑎𝑛𝑠^,5^𝑐𝑖𝑠^-diCQA (mono-cis), and **D**) 3^𝑐𝑖𝑠^,5^𝑐𝑖𝑠^-diCQA (di-cis).


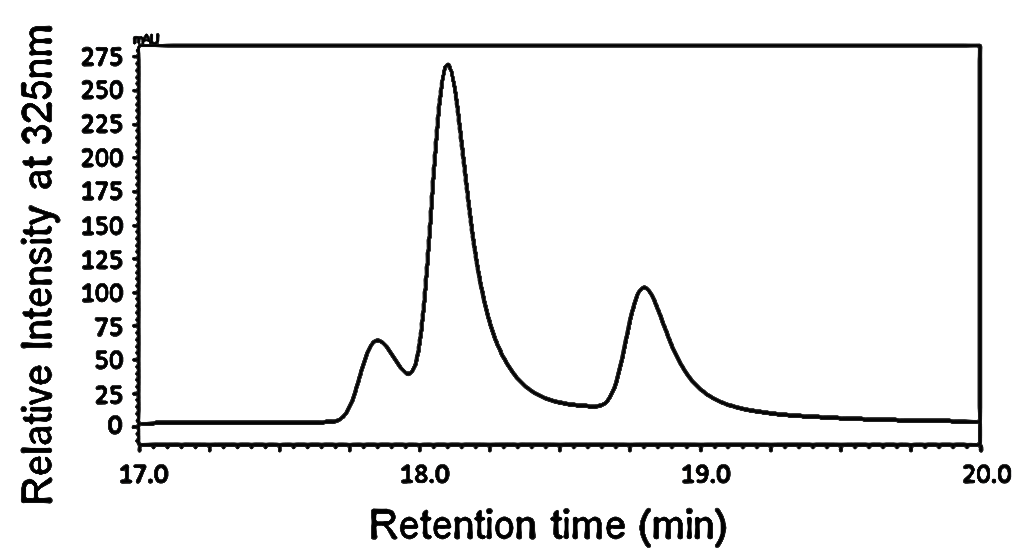


**Fig. S1.** Elution profile of 3,4-diCQA geometrical isomers obtained by UV-irradiation, using Phenomenex phenyl-hexyl and acetonitrile as the mobile phase composition.


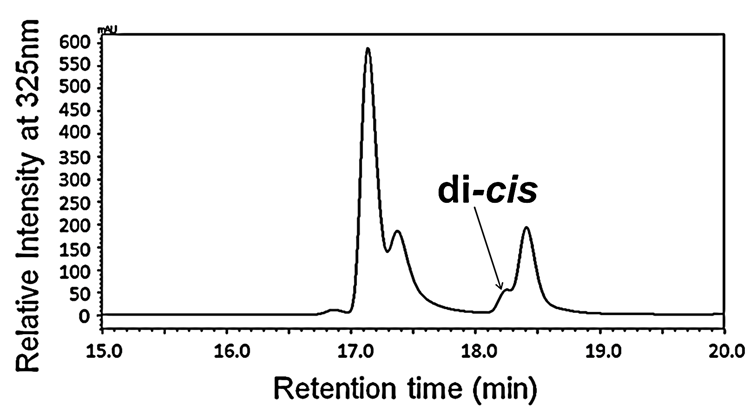


**Fig. S2.** Elution profile of 4,5-diCQA geometrical isomers obtained by UV-irradiation, using Phenomenex bi-phenyl and acetonitrile as the mobile phase composition.


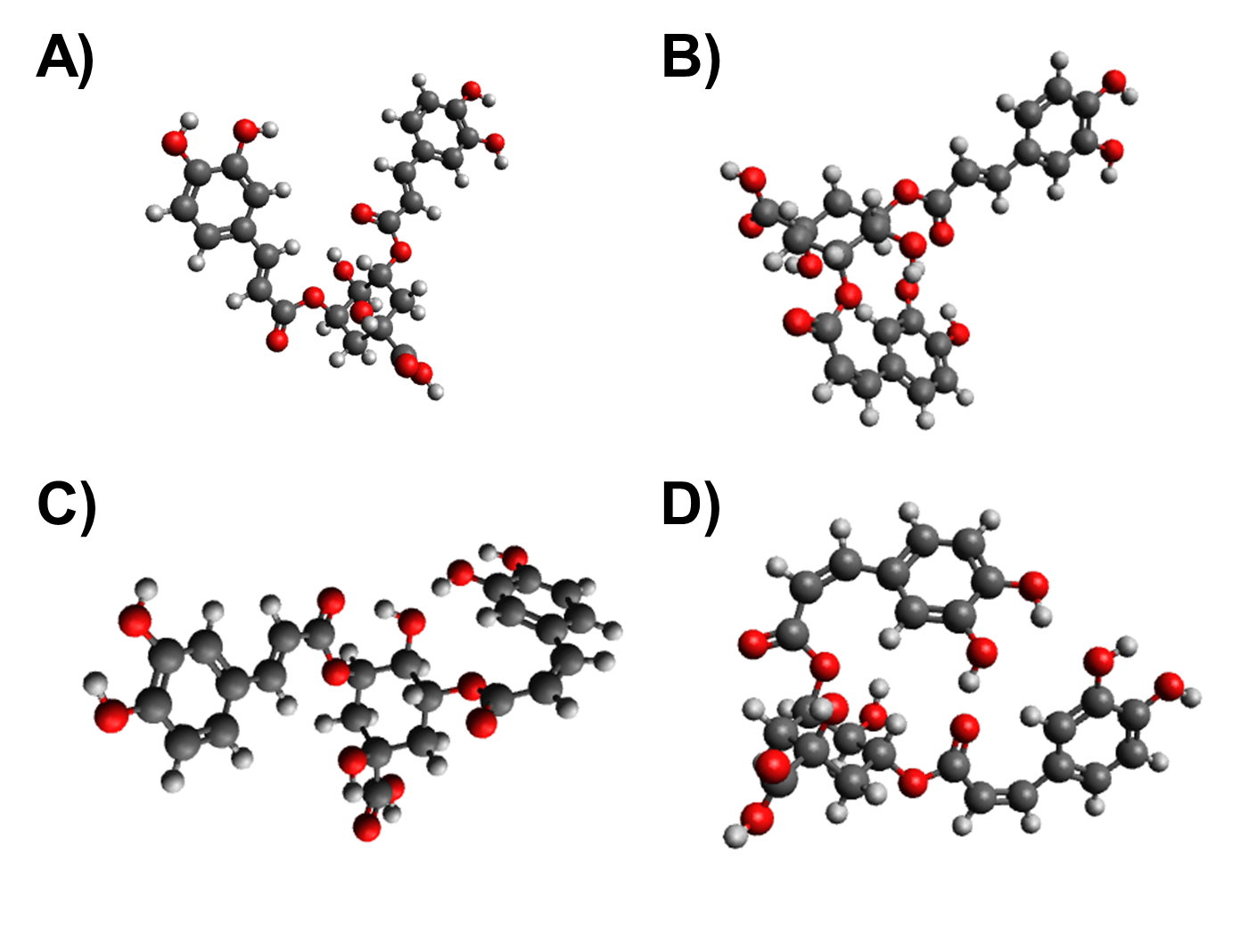


**Fig. S3.** Energy optimized structure of geometrical isomers of 3,5 diCQA. The structures were optimized using the B3LYP/6-311+G(d,p) method in Makola et al. [11]. **A**) 3^𝑡𝑟𝑎𝑛𝑠^,5^𝑡𝑟𝑎𝑛𝑠^-diCQA (di-trans), **B**) 3^𝑐𝑖𝑠^,5^𝑡𝑟𝑎𝑛𝑠^-diCQA (mono-cis), **C**) 3^𝑡𝑟𝑎𝑛𝑠^,5^𝑐𝑖𝑠^-diCQA (mono-cis), and **D**) 3^𝑐𝑖𝑠^,5^𝑐𝑖𝑠^-diCQA (di-cis).
